# Supplementary material for: Aquaculture of Animal Species: Their Eukaryotic Parasites and the Control of Parasitic Infections
Source: Biology (Basel). 2024 Jan 11;13(1):41. doi: 10.3390/biology13010041 (PMC10813438; doi:10.3390/biology13010041)
Supplement: Supplementary file 1 [file biology-13-00041-s001.zip › biology-2768193-supplementary.pdf]

Supplementary materials

Table S1. Selected references for various combinations of parasite taxa and important aquacultured species. (Supports Table 2)

	Taxa raised in aquaculture								
**Parasites**	**Corals**	**Annelida**	**Gastropoda**	**Bivalvia**	**Cephalopoda**	**Crustacea**	**Pisces**	**Anurans**	**Turtles**
**Protista**									
Protozoa			[1]	[1-3]		[2]	[4, 5]	[6, 7]	
Amoebae			[8, 9]					[10]	
Ciliates		[11]	[8]	[3, 12, 13]	[14]	[15, 16]	[17, 18]	[19]	
Flagellates		[8]	[8]				[17, 20, 21]		
Dinoflagellata				[22]		[9]	[17, 23, 24]		
Alveolata			[1]	[1, 12, 25]	[14, 26]			[19]	
Cercozoans				[1, 12, 22, 27-29]		[30]			[31]
Agregata					[20]	[20]			
Ascetosporea				[22]					
**Fungi, etc.**									
Oomycetes						[32, 33]	[4, 32, 34]	[32]	
Microsporidia			[35, 36]	[3, 35]	[32]	[15, 33]	[4, 37]		
Other			[8, 38]	[15, 22, 38]	[14]	[15, 39] [33]	[4, 17, 40, 41]		
**Plantae**									
Green algae?			[42]	[42, 43]		[33]	[40, 42]		[42]
Other			[8]						
**Animalia**									
Porifera				[3, 22]					
Cnidaria				[3]					
Myxozoa		[11, 44, 45]					[4, 46, 47]	[20]	
Acoela	[48]								
Acanthocephala					[26]	[33]	[4]		
Rhombozoa					[14]				
Annelida				[49]			[4, 50]		
Branchiura					[3]				
Citellata				[51]		[33]			
Polychaetes				[15, 17, 49, 51-53]		[33]			
Oligochaetes			[13]			[33]			
Hirudinea			[8, 13, 54]	[17]			[37]		
Nematoda			[8, 55, 56]	[3, 12, 22]	[14, 57]	[16, 33]	[4, 58, 59]	[6, 60, 61]	
**Platyhelminthes**									
Polycladidia	[48]								
Turbellaria			[8]	[3, 62]		[33]			
Monogenea					[14, 20]	[20]	[5, 63, 64]	[20]	[20]
Digenea	[48]	[11, 65]	[8, 65, 66]	[12, 22, 67]	[14, 57]	[33]	[17, 64, 68]	[61, 69]	
Cestoda		[11]		[3, 12, 22]	[14]	[16, 33]	[4, 17, 64]	[17]	
Temnocephalidae						[33]			
Nemertinea				[3]					

**Mollusca**				[3]				
Gastropoda	[48, 70, 71]	[3]	[72]	[3, 73, 74]				
Bivalvia							[75-78]	
**Arthropoda**	[48]						[4, 79]	[79]
Decapoda				[3, 22, 62]				
Copepoda, Ostracoda, Isopoda	[48, 80]	[80]	[3, 8, 80]	[3, 12, 22, 62, 80]	[14, 57, 80]	[33, 80]	[17, 77, 80, 81]	[79]
Branchiura					[14, 57]		[77]	
Cirripedia	[48]			[48]				
Acarina Mites				[13, 82]		[33]	[77]	
**Pisces**							[83, 84]	

References

1. Corbeil, S. and F.C.J. Berthe, *Disease and mollusc quality*, in *Shellfish Safety and Quality*. 2009. p. 270-294.
2. Berthe, F.C.J., et al., *Marteiliosis in molluscs: A review.* Aquatic Living Resources, 2004. **17**(4): p. 433-448.
3. Cheng, T., *Parasites of commercially important marine molluscs.* Advances in marine biology. New York: Academic, 1967: p. 199-261.
4. Noga, E.J., *Fish Disease. Diagnosis and Treatment.* 2010: Wiley-Blackwell. 519.
5. Shafiq, A., et al., *Parasite Diversity in a Freshwater Ecosystem.* Microorganisms, 2023. **11**(8).
6. Hernandez-Valdivia, E., et al., *Gastrointestinal parasites in bullfrogs (Lithobates catesbeianus) in aquaculture production units in the Mexican central highlands.* Rev Bras Parasitol Vet, 2023. **32**(2): p. e001523.
7. Kudo, R., *On the protozoa parasitic in frogs.* Transactions of the American Microscopical Society, 1922. **41**(2): p. 59-76.
8. O'Brien, M.F. and S. Pellett, *Diseases of Gastropoda.* Front Immunol, 2021. **12**: p. 802920.
9. Bradbury, P.C., *Parasitic Protozoa of Molluscs and Crustacea*, in *Parasitic Protozoa*. 1994. p. 139-264.
10. Weisbrod, T.C., et al., *Gastrointestinal entamoebiasis in captive anurans in North America.* Dis Aquat Organ, 2021. **143**: p. 109-118.
11. Stroud, J.L., *Diseases of annelids*, in *Invertebrate Pathology*. 2022. p. 163-170.
12. Jones, J.B. and J. Creeper, *Diseases of Pearl Oysters and Other Molluscs: A Western Australian Perspective.* Journal of Shellfish Research, 2006. **25**(1): p. 233-238.
13. McElwain, A., *Are parasites and diseases contributing to the decline of freshwater mussels (Bivalvia, Unionida)?* Freshwater Mollusk Biology and Conservation, 2019. **22**(2): p. 85-89.
14. Gestal, C., et al., *Handbook of pathogens and diseases in cephalopods*. 2019: Springer.
15. Chong, R.S.-M., *General introduction to pathophysiology of finfish, crustacea, and mollusks*, in *Aquaculture Pathophysiology*. 2022. p. 49-71.
16. Dominguez-Machin, M.E., et al., *Survey of protozoan, helminth and viral infections in shrimp Litopenaeus setiferus and prawn Macrobrachium acanthurus native to the Jamapa River region, Mexico.* Dis Aquat Organ, 2011. **96**(2): p. 97-103.
17. Hecht, T. and F. Endemann, *The impact of parasites, infections and diseases on the development of aquaculture in sub-Saharan Africa.* Journal of Applied Ichthyology, 1998. **14**(3-4): p. 213-221.
18. Bradbury, P.C., *Ciliates of Fish*, in *Parasitic Protozoa*. 1994. p. 81-138.
19. de Jager, G.P., L. Basson, and J. van Marwijk, *A New Trichodina Species (Peritrichia: Mobilida) from Anuran Tadpole Hosts, Sclerophrys spp. in the Okavango Panhandle, Botswana, with Comments on this Taxon.* Acta Protozoologica, 2019. **58**(3): p. 141-153.
20. Paladini, G., et al., *Parasitic Diseases in Aquaculture: Their Biology, Diagnosis and Control*, in *Diagnosis and Control of Diseases of Fish and Shellfish*. 2017. p. 37-107.
21. Woo, P.T.K., *Flagellate Parasites of Fish*, in *Parasitic Protozoa*. 1994. p. 1-80.
22. *Diseases and parasites*, in *Marine Bivalve Molluscs*. 2015. p. 429-477.
23. Jacobs, D.L., *A new parasitic dinoflagellate from fresh-water fish.* Transactions of the American Microscopical Society, 1946. **65**(1): p. 1-17.
24. Hoffman, G.L., H. Bishop, and C. Dunbar, *Algal parasite in fish.* The Progressive Fish-Culturist, 1960. **22**(4): p. 180-180.
25. Soudant, P., E.C. FL, and A. Volety, *Host-parasite interactions: Marine bivalve molluscs and protozoan parasites, Perkinsus species.* J Invertebr Pathol, 2013. **114**(2): p. 196-216.
26. Vidal, E.A., et al., *Cephalopod culture: current status of main biological models and research priorities.* Adv Mar Biol, 2014. **67**: p. 1-98.

27. Arzul, I. and R.B. Carnegie, *New perspective on the haplosporidian parasites of molluscs.* J Invertebr Pathol, 2015. **131**: p. 32-42.
28. Carrasco, N., T. Green, and N. Itoh, *Marteilia spp. parasites in bivalves: A revision of recent studies.* J Invertebr Pathol, 2015. **131**: p. 43-57.
29. Lynch, S.A., et al., *Detection of haplosporidian protistan parasites supports an increase to their known diversity, geographic range and bivalve host specificity.* Parasitology, 2020. **147**(5): p. 584-592.
30. Stentiford, G.D., et al., *Haplosporidium littoralis sp. nov.: a crustacean pathogen within the Haplosporida (Cercozoa, Ascetosporea).* Dis Aquat Organ, 2013. **105**(3): p. 243-52.
31. Mendonca, M.A., et al., *Detection of Hemopathogens in Chelonoidis carbonaria: Microscopic, Molecular, Hematological, and Clinical Biochemistry Aspects.* Vector Borne Zoonotic Dis, 2023. **23**(10): p. 520-527.
32. van den Berg, A.H., et al., *The impact of the water moulds <i>Saprolegnia diclina</i> and <i>Saprolegnia parasitica</i> on natural ecosystems and the aquaculture industry.* Fungal Biology Reviews, 2013. **27**(2): p. 33-42.
33. Edgerton, B.F., et al., *Synopsis of freshwater crayfish diseases and commensal organisms.* Aquaculture, 2002. **206**(1-2): p. 57-135.
34. Sandoval-Sierra, J.V., et al., *Saprolegnia species affecting the salmonid aquaculture in Chile and their associations with fish developmental stage.* Aquaculture, 2014. **434**: p. 462-469.
35. Carella, F. and G. De Vico, *Pathology, epidemiology, and phylogeny of mussel egg disease due to the microsporidian Steinhausia mytilovum (Field, 1924) in the Mediterranean mussel (Mytilus galloprovincialis).* J Invertebr Pathol, 2023. **198**: p. 107927.
36. Elizabeth McClymont, H., et al., *Molecular data suggest that microsporidian parasites in freshwater snails are diverse.* Int J Parasitol, 2005. **35**(10): p. 1071-8.
37. Jithendran, K., *Parasites and Parasitic Diseases in Fish Culture System*. 2014, Not Available.
38. Czeczuga, B., *Zoosporic fungi growing on freshwater molluscs.* Polish Journal of Environmental Studies, 2000. **9**(3): p. 151-156.
39. Song, T., et al., *Common disease-discriminatory fungal taxa accurately diagnose shrimp white feces syndrome, black gills, and retardation diseases.* Aquaculture, 2022. **561**.
40. Zhou, A., et al., *Interaction of environmental eukaryotic microorganisms and fungi in the pond-cultured carps: new insights into the potential pathogenic fungi in the freshwater aquaculture.* Environ Sci Pollut Res Int, 2021. **28**(29): p. 38839-38854.
41. Levy, M.G., et al., *Piscinoodinium, a fish-ectoparasitic dinoflagellate, is a member of the class Dinophyceae, subclass Gymnodiniphycidae: convergent evolution with Amyloodinium.* Journal of Parasitology, 2007. **93**(5): p. 1006-1015.
42. Vinyard, W. *Epizoophytic algae from mollusks, turtles, and fish in Oklahoma*. in *Proceedings of the Oklahoma Academy of Science*. 1953.
43. Zhao, L., et al., *New insight into light-enhanced calcification in mytilid mussels, Mytilus sp., infected with photosynthetic algae Coccomyxa sp.: δ13C value and metabolic carbon record in shells.* Journal of Experimental Marine Biology and Ecology, 2019. **520**.
44. Yokoyama, H., D. Grabner, and S. Shirakashi, *Transmission biology of the Myxozoa.* Health and environment in aquaculture, 2012: p. 3-42.
45. Okamura, B., A. Gruhl, and J.L. Bartholomew, *An introduction to myxozoan evolution, ecology and development*. 2015: Springer.
46. Blaylock, R.B. and S.A. Bullard, *Counter-insurgents of the blue revolution? Parasites and diseases affecting aquaculture and science.* J Parasitol, 2014. **100**(6): p. 743-55.
47. Kent, M.L., et al., *Recent advances in our knowledge of the Myxozoa.* J Eukaryot Microbiol, 2001. **48**(4): p. 395-413.
48. Barton, J.A., et al., *Parasites and coral‐associated invertebrates that impact coral health.* Reviews in Aquaculture, 2020. **12**(4): p. 2284-2303.
49. Sato-Okoshi, W., et al., *Polydorid species (Annelida: Spionidae) associated with commercially important oyster shells and their shell infestation along the coast of Normandy, in the English Channel, France.* Aquaculture International, 2022. **31**(1): p. 195-230.
50. ÖKtener, A. and N. Arslan, *A general review of parasitic Annelida (Hirudinea) recorded from different habitats and hosts in Turkey.* Turkish Journal of Zoology, 2012.

51. Tan, K., et al., *Research progress of shell boring mud-blister worm infestation in shellfish aquaculture.* Aquaculture, 2023. **574**.
52. Martinelli, J.C., et al., *Evaluating treatments for shell-boring polychaete (Annelida: Spionidae) infestations of Pacific oysters (Crassostrea gigas) in the US Pacific Northwest.* Aquaculture, 2022. **561**.
53. Rodewald, N., R. Snyman, and C.A. Simon, *Worming its way in-Polydora websteri (Annelida: Spionidae) increases the number of non-indigenous shell-boring polydorin pests of cultured molluscs in South Africa.* Zootaxa, 2021. **4969**(2): p. 255279.
54. Mack, J.M., et al., *Cryptic carnivores: Intercontinental sampling reveals extensive novel diversity in a genus of freshwater annelids.* Mol Phylogenet Evol, 2023. **182**: p. 107748.
55. Morley, N.J., *Aquatic molluscs as auxiliary hosts for terrestrial nematode parasites: implications for pathogen transmission in a changing climate.* Parasitology, 2010. **137**(7): p. 1041-56.
56. Grewal, P., et al., *Parasitism of molluscs by nematodes: types of associations and evolutionary trends.* Journal of nematology, 2003. **35**(2): p. 146.
57. Iglesias, J., L. Fuentes, and R. Villanueva, *Cephalopod culture*. 2014: Springer Science & Business Media.
58. Eiras, J.C., et al., *An Overview of Fish-borne Nematodiases among Returned Travelers for Recent 25 Years- Unexpected Diseases Sometimes Far Away from the Origin.* Korean J Parasitol, 2018. **56**(3): p. 215-227.
59. Bakenhaster, M.D., et al., *Philometra floridensis (Nematoda: Philometridae) damages ovarian tissue without reducing host (Sciaenops ocellatus) fecundity.* Dis Aquat Organ, 2014. **108**(3): p. 227-39.
60. Bursey, C.R. and D.R. Brooks, *Nematode Parasites of 41 Anuran Species from the Area de Conservación Guanacaste, Costa Rica.* Comparative Parasitology, 2010. **77**(2): p. 221-231.
61. Chikhlyaev, I.V. and A.B. Ruchin, *Helminths of amphibians (Amphibia) in beaver ponds in the Central Russia.* Aquaculture, Aquarium, Conservation & Legislation, 2020. **13**(6): p. 3810-3821.
62. Sanil, N. and K. Vijayan, *Diseases and Parasites of Bivalves.* 2011.
63. Hoai, T.D., *Reproductive strategies of parasitic flatworms (Platyhelminthes, Monogenea): the impact on parasite management in aquaculture.* Aquaculture International, 2019. **28**(1): p. 421-447.
64. Bautista-Hemández, C.E., et al., *Helminth communities of Xiphophorus malinche (Pisces: Poeciliidae), endemic freshwater fish from the Pánuco River, Hidalgo, Mexico.* Revista Mexicana de Biodiversidad, 2014. **85**(3): p. 838-844.
65. Ditrich, O., et al., *Larval stages of trematodes from freshwater molluscs the Yucatan Peninsula, Mexico.* Folia Parasitologica, 1997. **44**(2): p. 109-127.
66. Barton, D.P., et al., *Parasites of Selected Freshwater Snails in the Eastern Murray Darling Basin, Australia.* Int J Environ Res Public Health, 2022. **19**(12).
67. Curran, S.S., R.D. Gonzales, and S.A. Bullard, *Molecular Characterization of Sporocysts and Cercariae (Digenea: Bucephalidae) Infecting the Eastern Oyster Crassostrea Virginica from Virginia.* J Parasitol, 2023. **109**(3): p. 259-263.
68. Pinto, H.A., et al., *Experimental and molecular study of cercariae of Clinostomum sp. (Trematoda: Clinostomidae) from Biomphalaria spp. (Mollusca: Planorbidae) in Brazil.* J Parasitol, 2015. **101**(1): p. 108-13.
69. Crotti, M., *Digenetic Trematodes: an existence as parasites. Brief general overview.* Microbiologia Medica, 2013. **28**(2).
70. Gittenberger, A. and B. Hoeksema, *Habitat preferences of 20 Indo-West Pacific wentletrap species (Gastropoda: Epitoniidae) associated with scleractinian corals*. 2006, Chapter.
71. Hoeksema, B. and A. Gittenberger, *Records of some marine parasitic molluscs from Nha Trang, Vietnam.* Basteria, 2008. **72**(4/6): p. 129-133.
72. Maguire, A.K. and L. Rogers-Bennett, *An ectoparasitic snail (Evalea tenuisculpta) infects red abalone (Haliotis rufescens) in northern California.* California Fish and Game, 2013. **99**(2): p. 80-89.
73. Cumming, R.L. and R.A. Alford, *Population dynamics of Turbonilla sp.(Pyramidellidae, Opisthobranchia), an ectoparasite of giant clams in mariculture.* Journal of experimental marine biology and ecology, 1994. **183**(1): p. 91-111.

74. Boglio, E. and J. Lucas, *Impacts of ectoparasitic gastropods on growth, survival, and physiology of juvenile giant clams (Tridacna gigas), including a simulation model of mortality and reduced growth rate.* Aquaculture, 1997. **150**(1-2): p. 25-43.

75. Horne, L., D. DeVries, and J. Stoeckel, *The Effects of Glochidia Infection on the Metabolic Rate and Hypoxia Tolerance of Bluegill Lepomis Macrochirus and Largemouth Bass Micropterus Salmoides.* J Parasitol, 2022. **108**(5): p. 467-475.

76. Chowdhury, M.M.R., T.J. Marjomäki, and J. Taskinen, *Effect of glochidia infection on growth of fish: freshwater pearl mussel Margaritifera margaritifera and brown trout Salmo trutta.* Hydrobiologia, 2019. **848**(12-13): p. 3179-3189.

77. Heckmann, R., *Other ectoparasites infesting fish: Copepods, branchiurans, isopods, mites and bivalves.* AQUACULTURE MAGAZINE-ARKANSAS-, 2003. **29**(6): p. 20-31.

78. Zieritz, A., et al., *Identifying freshwater mussels (Unionoida) and parasitic glochidia larvae from host fish gills: a molecular key to the North and Central European species.* Ecol Evol, 2012. **2**(4): p. 740-50.

79. Kupferberg, S.J., et al., *Parasitic Copepod (Lernaea cyprinacea) Outbreaks in Foothill Yellow-legged Frogs (Rana boylii) Linked to Unusually Warm Summers and Amphibian Malformations in Northern California.* Copeia, 2009. **2009**(3): p. 529-537.

80. Williams, E.H. and L. Bunkley-Williams, *Life Cycle and Life History Strategies of Parasitic Crustacea*, in *Parasitic Crustacea*. 2019. p. 179-266.

81. Mic, R., E. Rehulkova, and M. Seifertova, *Species of Ergasilus von Nordmann, 1832 (Copepoda: Ergasilidae) from cichlid fishes in Lake Tanganyika.* Parasitology, 2023. **150**(7): p. 579-598.

82. Edwards, D.D. and M.F. Vidrine, *Host Diversity Affects Parasite Diversity: A Case Study Involving Unionicola spp. Inhabiting Freshwater Mussels.* J Parasitol, 2020. **106**(5): p. 675-678.

83. Silva, S., et al., *The haematophagous feeding stage of anadromous populations of sea lamprey Petromyzon marinus: low host selectivity and wide range of habitats.* Hydrobiologia, 2014. **734**(1): p. 187-199.

84. Salinger, J.M. and R.L. Johnson, *Parasitism of Rainbow Trout in Hatchery Raceways by Chestnut Lampreys.* North American Journal of Aquaculture, 2019. **81**(3): p. 230-234.
